# Spontaneous Premature Ovarian Insufficiency: Methods Under Research for Infertility Treatment

**DOI:** 10.3390/jcm15093224

**Published:** 2026-04-23

**Authors:** Ranko Kutlesic, Marija Kutlesic, Jelena Milosevic-Stevanovic, Predrag Vukomanovic, Danka Mostic-Stanisic

**Affiliations:** 1 Department of Gynaecology and Obstetrics, Faculty of Medicine, University of Nis, 18000 Nis, Serbia; 2 Clinic of Gynaecology and Obstetrics, University Clinical Centre Nis, 18000 Nis, Serbia; 3 Institute of Gynaecology and Obstetrics Belgrade, University Clinical Centre of Serbia, 11000 Belgrade, Serbia; 4 Faculty of Medicine, University of Belgrade, 11000 Belgrade, Serbia

**Keywords:** reproductive endocrinology, infertility, premature ovarian insufficiency

## Abstract

Premature ovarian insufficiency (POI) is a clinical condition characterized by loss of ovarian function indicated by amenorrhea or irregular menstrual cycles for at least 4 months and elevated gonadotrophins (FSH > 25 IU/L, measured on one occasion) and low estrogen serum levels in women under the age of 40. Premature ovarian insufficiency can be non-iatrogenic or spontaneous (idiopathic or due to genetic, autoimmune, or metabolic reasons, or infections) and iatrogenic (a consequence of oophorectomy, chemotherapy, radiotherapy, or uterine artery embolization). Women with POI are faced not only with estrogen deficiency but also with infertility and psychological implications. Hormonal replacement therapy is effective in treating the symptoms of premature ovarian insufficiency as well as in lowering the health risk of long-term consequences of premature ovarian insufficiency. Currently, oocyte donation is the standard treatment for patients with POI desiring pregnancy. Recently developed methods for the regeneration of ovarian tissue, such as stem cell therapy, platelet-reach plasma therapy and in vitro activation of ovarian tissue, are still under research and further adequate multicentric clinical studies are needed to develop standardized effective and safe protocols for the infertility treatment of patients with premature ovarian insufficiency.

## 1. Introduction

Premature ovarian insufficiency (POI) is a clinical condition characterized by loss of ovarian function due to depletion of ovarian follicles, indicated by amenorrhea or irregular menstrual cycles, elevated gonadotrophins and low estrogen serum levels in women under the age of 40 [1], and the condition was first described by Fuller Albright in 1942 [2]. Patients with POI suffer from symptoms associated with estrogen deficiency, as well as from infertility, impaired bone and cardiovascular health, and sexual and neurological function. POI has a significant impact on psychological well-being and quality of life.

More precisely, such patients have amenorrhoea or irregular menstrual cycles for at least 4 months and elevated serum FSH levels > 25 IU/L measured on one occasion (FSH testing for diagnosing POI should not be limited to a specific day of the menstrual cycle). In cases with diagnostic uncertainty, FSH measurements should be repeated after 4–6 weeks [3,4,5,6]. Estradiol serum measurements alone are not sufficient for diagnosing POI, but low estradiol serum concentrations in combination with elevated FSH concentrations additionally confirm the diagnosis of POI [6]. In fact, POI is the state of hypergonadotropic hypogonadism, which could manifest as primary amenorrhea (with onset before the age of menarche) or as secondary amenorrhea (i.e., cessation of menstrual cycles in a woman who had menstrual cycles in the past). These ovaries have functioned, but stopped functioning before the usual age of menopause. According to the ESHRE guideline [6] the term premature ovarian insufficiency is more adequate than failure, “as this more accurately describes the fluctuating nature of the condition and does not carry the negative connotation of “failure” [6].” Women with POI are faced not only with symptoms of estrogen deficiency but also with infertility and the impact of POI on psychological wellbeing.

A few decades ago, the incidence of premature ovarian failure was estimated to be 0.1% in women <30 years and 1% in women under the age of 40. In younger populations POI affects 1/10,000 women by the age of 20 [7,8]. A recent systematic review and meta-analysis, based on 13 studies in 10 countries, reported that the global prevalence of premature ovarian insufficiency is 3.5%, but there are regional differences: the highest prevalence is in North America (11.3%), followed by South America (5.4%), Asia (3.3%) and Europe (2.3%), with a rising (although not significant) trend over the 20 past years. According to that study, the prevalence of premature ovarian insufficiency is 5.3% in a developing country and 3.1% in a developed country [9]. On the other hand, another study reported that the likelihood of getting diagnosed with premature ovarian insufficiency under the age of 40 in the Finnish female population is about 0.5% [10], and in Sweden the prevalence was found to be 1.9% [11]. Such significant heterogeneity regarding the prevalence of POI among the studies and geographical regions may be due to factors encompassing multiple etiologies in different geographic regions as well as different study qualities. The reported higher prevalence rate of POI in North America may be due to the fact that the US studies included women with iatrogenic POI after chemo/radiotherapy. Moreover, the authors of this meta-analysis reported a difference in POI prevalence between high-quality studies (2.8%) and low-quality studies (10.2%). This may be the consequence of different types of studies: high-quality articles were all cohort studies with large sample sizes, and low-quality articles were all cross-sectional studies with small sample sizes [9].

Premature ovarian insufficiency can be non-iatrogenic or spontaneous (idiopathic or due to genetic, autoimmune, metabolic reasons, FSH receptor mutations, or infections) and iatrogenic—a consequence of oophorectomy, chemotherapy, radiotherapy, or uterine artery embolization. (Table 1). The etiology of non-iatrogenic premature ovarian insufficiency is complex and heterogenic, being unknown in most of the cases. A number of cases of ovarian insufficiency have been reported to be a result of autoimmune disorders such as autoimmune thyroid diseases, adrenal insufficiency, type 1 diabetes, etc.

Idiopathic ovarian insufficiency is associated with numerous mechanisms which are not fully understood and is connected with more than 70 genes which play a role in ovarian development and function, including gonadogenesis, follicle and oocyte maturation, meiosis and ovulation [12]. It was also reported that the incidences of known reasons for premature ovarian insufficiency are as follows: genetic—chromosomal abnormalities in 10–13% of cases, autoimmune in 4–30% of cases, iatrogenic in 7.6–15.8% of cases [13,14,15]. These percentages were questioned by a recent study [16], where the authors stated that the etiological factors for the development of premature ovarian insufficiency are as follows: genetic in 9.9% of cases, autoimmune in 18.9% of cases, iatrogenic in 34.2% of cases, and idiopathic in 36.9% of cases. The reason for such changes is the improvement of diagnostic capabilities.

## 2. Materials and Methods

An extensive literature search was conducted using MEDLINE via PubMed and Google Scholar databases covering the period from 1 January 1980 to 1 June 2025. In order to identify the articles regarding premature ovarian insufficiency, the search was particularly focused on the current treatments of infertility in patients with POI. Two reviewers (RK and MK) independently searched through the literature using the terms “premature ovarian insufficiency” [Mesh] and “Infertility” [Mesh]. The primary aim was to present clinical studies on novel methods of infertility treatment in patients with premature ovarian insufficiency. The secondary aim was to present current knowledge on etiology, pathogenesis, clinical presentation and the treatment of non-iatrogenic or spontaneous premature ovarian insufficiency. The exclusion criteria applied included a lack of full-text access through our resources, so a total of 125 articles were included in this review.

No generative artificial intelligence (GenAI) has been used in this paper to generate text, data, or graphics, or to assist in study design, data collection, analysis, or interpretation.

## 3. Molecular Biological Mechanisms in Development of POI

There are two hypotheses for the development of spontaneous (non-iatrogenic) premature ovarian insufficiency: inadequate primordial follicle pool or accelerated depletion of oocytes and follicles [17]. Accelerated apoptosis in granulosa cells is also a factor responsible for extensive follicular atresia [18].

Recent studies have reported the possible mechanisms, focused on DNA and RNA, that could lead to such conditions. Human ovaries contain follicles, where the gamete—oocyte—is surrounded by follicular cells. During follicular maturation, intense proliferation of follicular granulosa cells happens and the capacity of follicular cells to proliferate is crucial for the quantity and quality of ovarian follicles. The proliferation capacity of cells depends on telomere length and telomerase activity. Telomere are nucleoprotein structures that cap chromosome extremities and telomere length shortens with each cell division, so telomere attrition is considered to be one of the hallmarks of aging [19].

Telomerase is a ribonucleic complex capable of compensating for telomere attrition by adding telomeric repeats to telomeres [20]. Telomerase activity is high in human ovaries, especially in granulosa cells, enabling their numerous replications during the follicologenesis. Recent studies on telomere length and telomerase activity have reported that shorter telomeres and lower telomerase activity in granulosa cells seem to be associated with premature occult ovarian insufficiency. In women, telomerase activity in ovaries declines with age and “ovarian aging” is dependent on telomerase activity in granulosa cells [21].

Premature ovarian failure and reproductive aging may be the consequences of accumulated DNA damage and deficient DNA repair capacity in granulosa cells and oocytes in humans [22]. Activation of the DNA damage system in aged oocytes and subsequent oocyte damage could be induced by inflammatory microenvironment in the ovary, so chronic inflammation is considered to be one of the potential confounding factors in ovarian aging and development of premature ovarian insufficiency [23].

Recent studies have focused on microRNAs (miRNAs) and their role in the pathophysiology of premature ovarian failure. MicroRNAs are a class of RNAs encoded in the mammalian genome that direct gene silencing of genes involved in the regulation of proliferation, differentiation, apoptosis, development, tumorigenesis, and hematopoiesis [24].

The authors of a recent study demonstrated that miR-379-5p overexpression inhibited granulosa cell proliferation and attenuated DNA repair efficiency and in patients with biochemical premature ovarian insufficiency miR-379-5p was expressed at higher levels compared with normal controls [25]. Other studies on microRNAs reported abnormal expression of miRNAs observed in plasma or blood from patients with POI, indicating that miRNAs may be non-invasive diagnostic tools in clinical practice [25,26]. Other noncoding RNAs and their role in the pathogenesis and treatment of premature ovarian insufficiency are also the subject of recent investigations.

## 4. Practical Approach to Premature Ovarian Insufficiency

### 4.1. Clinical Presentation of Premature Ovarian Insufficiency

In most patients, cycle disturbances and subfertility are the first signs of premature ovarian insufficiency. The initial trigger for the disease is unknown. Primary amenorrhoea is present in 15.7% and secondary amenorrhoea is present in 84.3% of patients with POI [27].

Amenorrhoea is not required for the diagnosis of POI [6]. The level of cycle disturbances depends on the clinical state of premature ovarian insufficiency: oligomenorrhoea is more frequent during occult and biochemical ovarian insufficiency.

Menstrual cycle irregularities may be followed by symptoms related to estrogen deficiency such as sweating, hot flashes, vaginal irritation and dryness, dyspareunia, urine urgency, loss of skin elasticity, dry eye syndrome, loss of hair, abnormal sleep pattern and mental health issues.

Psychological implications of premature ovarian insufficiency should not be underestimated, particularly the consequences of impaired sexual function: these may include associated anxiety, depressed mood, and impaired interactions with their peers, which leads to feeling less feminine and having decreased self-esteem [28].

The other symptoms are also reported in patients with premature ovarian insufficiency such as mood swings and mental fog (>75% reporting), cold intolerance and joint clicking (>50% reporting), tingling in the limbs (33%), low blood pressure (∼33%); hypothyroidism (17%), hypoglycemia (16%) and gluten allergies (10%) [29].

The clinical presentation of premature ovarian insufficiency is characteristic and quite suggestive. However, there could be differences between individual cases as well as in the severity of symptoms. Patients with premature ovarian insufficiency could experience more severe symptoms compared with women with natural menopause. Moreover, the intensity of symptoms may vary in the same patient, depending on variation in hormone production, which is characteristic for spontaneous premature ovarian insufficiency. It is important to determine the age of the patient, since classically premature insufficiency was diagnosed in women under the age of 35 years [30] or in patients younger than 40 years, according to other guidelines [6].

### 4.2. Diagnostic Work-Up in Patients with Premature Ovarian Insufficiency

The diagnosis of POI should be made based on clinical presentation, the presence of spontaneous amenorrhea or irregular menstrual cycles, and biochemical confirmation [6].

Family history should also be examined as female relatives (such as sisters or daughters) of women with non-iatrogenic POI are at increased risk of developing POI themselves. Family history should be checked for the presence of male intellectual disability (suggesting fragile X syndrome). The frequency of gene mutations in patients with POI differs between populations and depends on the enrichment of mutations for certain recessively inherited disorders due to population bottlenecks and genetic drift in the genetically isolated populations [31].

In clinical practice, genetic investigations are not often performed, mainly because of the cost of sequencing individual genes and the fact that most genes account for only a small portion of patients with POI. However, clinicians should be aware of POI genetics and perform such investigations in patients with POI with eyelid malformations or hearing loss. In some cases, hearing abnormalities could be mild and it may be difficult for reproductive specialists to recognize them [11].

The clinician should look for clinical signs of thyroid disease (the presence of goiter, exophthalmos, bradycardia or tachycardia, cold and dry or soft and warm skin, as well as for clinical signs of adrenal insufficiency (orthostatic hypotension, hyperpigmentation, and decreased axillary and pubic hair). Other signs of autoimmune disease may include vitiligo (often associated with thyroid and adrenal autoimmunity), nail dystrophy and mucocutaneous candidiasis in autoimmune polyglandular syndrome type 1, alopecia areata and malar rash (in SLE).

Diagnosis is confirmed with serum FSH, AMH and estradiol levels. In cases with diagnostic uncertainty the diagnosis of premature ovarian insufficiency should be confirmed with elevated serum FSH levels measured on two separate occasions, 4–6 weeks apart [6]. The threshold of FSH elevation differs among guidelines: from >25 IU/L [6] or >30 IU/L [27] to over 40 IU/L [32]. It is a mistake to use elevated serum FSH levels alone to diagnose irreversible premature ovarian insufficiency: the results of serum FSH measurements should be interpreted along with clinical presentation [33] or in combination with estradiol and serum AMH levels. It is important to repeat the test if serum FSH levels are in the menopausal range. According to new recommendations FSH testing for the diagnosis of POI does not have to be timed to a specific day of the menstrual cycle [6].

At the same time estrogen serum levels should be measured to confirm hypogonadism. Estradiol serum levels less than 50 pmol/L are part of the diagnosic criteria for premature ovarian insufficiency [32,34]. Low estradiol serum levels indicate hypoestrogenism, but the diagnosis of POI should not be based only on low estradiol concentrations: the combination of low estradiol and elevated serum FSH levels provide confirmation of premature ovarian insufficiency [6]. The progesterone withdrawal test is not a substitute for serum FSH measurement because about 50% of women with premature ovarian insufficiency will respond to a progestogen challenge test due to intermittent ovarian function and intermittent hypoestrogenism which is the unique characteristic of premature ovarian insufficiency and the main difference from a definitive state of early menopause [35].

Anti-Mullerian hormone (AMH) is a peptide produced by small antral follicles and reflects a number of developing antral follicles, which is currently thought to be the most reliable measure of impaired ovarian reserve. Serum AMH levels remain stable throughout the menstrual cycle. Serum concentrations of AMH strongly correlate with the number of viable follicles remaining and developing in the ovaries [36] and progressively decline as women approach menopause [37]. Nevertheless, the diagnostic cut-off for serum AMH levels is not established because AMH could be undetectable a few years before the cessation of menstruation [38]. Serum AMH levels correlate with antral follicle count (AFC) and ovarium volume on a transvaginal ultrasound scan, although in some patients antral follicle count could be normal in association with low AMH. It was recommended that AMH testing may be useful to confirm a POI diagnosis where FSH results are inconclusive, but AMH results need to be interpreted within the clinical context. AMH testing should not be performed to predict premature ovarian insufficiency due to insufficient evidence of accuracy [6]. In most patients with POI serum AMH levels are low [39]. From the clinical point of view, serum AMH levels depend on the clinical state of POI and reflect the gradual loss of ovarian follicles, declining from occult to biochemical to overt POI. The importance of AMH as a measure of follicular reserve is related to the role of AMH in follicular growth and differentiation. AMH secretion peaks at the stage when the follicle becomes selectable—the preantral stage in mice and the antral stage in humans. During the gonadotropin-independent phase of follicular growth AMH inhibits the recruitment of primordial follicles into the pool of growing follicles and delays the growth of follicles. AMH inhibits premature differentiation of granulosa cells and reduces their sensitivity to FSH. During the gonadotropin-dependent phase of follicular growth AMH plays a role in the selection of follicles by decreasing the responsiveness of certain growing follicles to FSH which ensures that the most viable follicles are selected for ovulation while others undergo atresia [40].

In patients with spontaneous premature ovarian insufficiency karyotype should be performed as well as testing for the FMR1 gene premutation, if available.

Autoantibody testing should also be performed in patients with premature ovarian insufficiency. The most sensitive test for adrenal cortex is the identification of 21-hydroxylase antibodies (21OH-Ab) in peripheral blood, and this analysis—or, alternatively, the identification of adrenocortical antibodies (ACA)—should be carried out in all patients with premature ovarian insufficiency. A test of adrenal function should be performed in patients with positive 21OH-Ab/ACA. Identification of thyroid peroxidase autoantibodies, thyroglobulin antibodies and thyroid function tests also should be performed in patients with premature ovarian insufficiency. Ovarian antibody testing could be carried out, but there is a weak correlation with clinical presentation and false positive results are possible [41]. Clinicians should take autoimmune diseases into consideration in patients with POI and implement appropriate screening. Patients with adrenal or thyroid hypofunction should be referred to an endocrinologist for adequate treatment [42].

### 4.3. Differential Diagnosis of Premature Ovarian Insufficiency

Patients with POI should be examined for the presence of other reasons for hypergonadotropic hypogonadism.

Resistant ovary syndrome (ROS) is characterized by hypergonadotropic hypogonadism and normal ovary reserve, with the presence of preantral and antral follicles which do not respond to endogenous human gonadotrophins [43]. The leading causes responsible for the development of resistant ovary syndrome are follicle-stimulating hormone receptor (FSHR) mutations and autoimmune disorders characterized by the presence of autoimmune antibodies against FSH receptors that inhibit FSH binding to its receptor [43]. The other potential causes of this disorder are mutations in the FSH beta subunit, abnormal gonadotropin signaling, and deficiency of follicle-stimulating growth factors [44]. The clinical presentation of ROS includes symptoms of POI, hypergonadotropism, hypoestrogenism, and decreased sensitivity even to high-dose gonadotropins. Serum AMH levels in patients with POI are lower than serum AMH levels in patients with resistant ovary syndrome. Clinical investigation, including transvaginal ultrasound examination and antral follicle count measurement, should definitely differentiate between POI and ROS patients. Making this distinction is important because of the potential for controlled ovarian stimulation and in vitro maturation of immature oocytes in ROS patients who desire pregnancy [43]. It is also important to note that gonadotropin resistant ovaries return to normal from time to time and should not be treated with hormonal substitution therapy suppressing ovulation.

FSH receptor inactivating mutations are an uncommon cause of sporadic POI [45], although some FSHR mutations have been described to be associated with premature ovarian insufficiency [46,47]. In fact, FSHR inactivating mutations have variable clinical presentations ranging from primary amenorrhea with pubertal abnormalities to secondary amenorrhea and premature menopause [46].

Polycystic ovary syndrome (PCOS) has also been associated with premature ovarian insufficiency. A recent population-based study of PCOS patients and women without PCOS reported higher risk for premature ovarian insufficiency in PCOS patients than in controls, with an even higher risk in women with PCOS who did not receive metformin treatment [48].

### 4.4. Hormonal and Non-Hormonal Treatment of Patients with POI

The hormone replacement therapy for premature ovarian insufficiency includes estrogen, progestogen and estrogen–progesterone combinations (continuous and sequential), as well as combined oral contraceptives. Administration of testosterone could be considered, but only for hypoactive sexual desire disorder [6]. The doses of hormones should be enough to replace deficiencies, i.e., to reach the serum levels as close as possible to natural hormone levels. Estrogen doses for patients with POI should be higher than those used in patients with natural menopause, regarding the younger age of patients with premature ovarian insufficiency, symptom relief, and desirable metabolic effects (increased HDL and decreased cholesterol levels), as well as reduced progression of subclinical atherosclerosis and long-term beneficial effects on cardiovascular and bone health [49,50,51,52]. The main risks of HRT are well known, including the development of breast cancer, endometrial cancer and thrombotic events.

Non-hormonal pharmacologic and non-pharmacologic therapies that are effective in peri-/postmenopausal women could be considered in patients with POI, but there is no specific evidence for POI. Similarly, specific evidence on lifestyle interventions in POI is also limited, but patients with POI should be encouraged to adopt a healthy lifestyle to improve their overall well-being. Patients with premature ovarian insufficiency should have a calcium-rich diet and vitamin D supplementation to prevent osteoporosis [6]. An intake of calcium (1200 mg daily) and vitamin D (at least 800 IU daily) is considered to be adequate for such patients [53,54].

Patients with POI considering using other nutrient supplements and herbal medicines should be informed that there is insufficient evidence to support their use [6].

Much of the current evidence on HRT is not gained from the studies conducted on patients with POI, but on studies involving women with physiological menopause or from non-randomized controlled studies. Adequate studies on patients with premature ovarian insufficiency are still lacking. In clinical practice, more physiological regimens should therefore be preferred, and synthetic estrogen-containing formulations should be avoided due to their long hepatic half-life and pro-thrombotic and hypertensive effects and less beneficial effects on bone and metabolism. HRT in patients with POI should be continued until the average age of natural menopause [6,54,55,56,57].

## 5. The Treatment of Infertility in Patients with POI

Hormone replacement therapy, changes in lifestyle and appropriate diet are successful treatments for estrogen deficiency in most patients, but treating infertility in patients with spontaneous premature ovarian insufficiency represents one of the greatest challenges in reproductive endocrinology.

Oocyte donation is the routine treatment for infertility in patients with premature ovarian insufficiency, but there are other treatment options such as stem cell therapy, platelet-rich plasma and primordial follicle activation as well as treatments optimizing the endometrial receptivity. Such options are still under investigation.

### 5.1. Oocyte Donation

Oocyte donation is a POI treatment that gives a real and good chance for couples to achieve pregnancy. The first successful human oocyte donations were performed in the 80s [58,59].

In an early report involving five fertilized oocytes that were recovered and transferred, two pregnancies were achieved; in both successful pregnancies, donor and recipient LH surges were synchronized [60]. Nowadays, oocyte donation has become a routine practice in assisted reproductive technologies (ART), and the pregnancy rate per fresh embryo transfer is reported to be 49.6% and per frozen embryo transfer 44.9% [61]. The success of egg donation is higher with younger donor age, but outcomes are independent of the recipient’s age [62]. Such pregnancies should be carefully monitored due to the risk of pregnancy complications, such as the risk of gestational hypertension, pre-eclampsia [63,64], diabetes mellitus [65], intrahepatic cholestasis, preterm labor, low and very low birth weight, and small-for-date infant [66].

### 5.2. Stem Cell Therapies

Stem cells are defined as clonogenic cells capable of self-renewal and multilineage differentiation. Pluripotent stem cells, i.e., embryonic stem cells or induced pluripotent stem cells, differentiate into cells of all three embryonic layers. Multipotent stem cells can develop into specialized cells in a specific tissue, like hematopoetic cells that can develop into all blood cell types, including red blood cells, white blood cells, and platelets. Unipotent cells differentiate only into one cell type. Stem cell therapy offers novel treatments for various cardiovascular, neurological, orthopedic and other conditions as well as in experimental treatments in conditions such as ovarian decline and premature ovarian insufficiency [67].

The most studied stem cells used for ovarian regenerative purposes are mesenchymal stem cells (MSC) and hematopoietic stem cells (HSC). The other type of stem cells also used for such purposes are adipose-derived stem cells [68].

Mesenchymal stem cells (MSCs) are multipotent stromal cells that can differentiate into osteoblasts, chondrocytes, myocytes and adipocytes. MSCs can be found in bone marrow, dental pulp, adipose tissue, umbilical cord and placenta [69,70,71,72], as well as in muscles, endometrium, and the uterine cervix [73,74,75].

MSCs have immunomodulatory and anti-inflammatory properties and secrete bioactive molecules, such as cytokine and growth factors, supporting tissue repair, angiogenesis and cell survival [76], making them a candidate for the treatment of premature ovarian insufficiency. Bioactive molecules such as VEGF, HGF and IGF-1 secreted by MSCs could stimulate angiogenesis and increase blood flow in ovarian tissue, which helps reduce apoptosis of ovarian cells. Such conclusions are derived from animal studies, e.g., studies showing that the transplantation of human umbilical cord stem cells could improve ovarian function in mouse models with cyclophosphamide-induced premature ovarian insufficiency [77].

The authors of non-randomized clinical trial performed transplantation of umbilical cord-derived mesenchymal stem cells to patients’ ovaries by orthotopic injection under the guidance of vaginal ultrasound in 61 patients with POI. The authors concluded that the transplantation rescued the ovarian function of patients with POI, as indicated by increased follicular development and improved egg collection. There were four deliveries, three after ICSI and one after naturally conception [78].

Hematopoietic stem cells (HSC) are involved in hematopoiesis and can be found in bone marrow, peripheral and umbilical cord blood, playing an essential role in immune regulation. Animal studies demonstrated that stem cells could improve hormone levels, follicle count, the estrous cycle and pregnancy outcomes [79].

The authors of a systematic review and meta-analysis including twenty animal and six clinical studies reported that the results of the clinical studies showed that stem cell treatment could decrease FSH levels (MD: −30.32, 95% CI −59.03 to −1.01, *p* = 0.04), increase antral follicle count (MD: 1.07, 95% CI 0.70–1.43, *p* < 0.00001), increase pregnancy rate (RD: 0.19, 95% CI 0.04–0.34, *p* = 0.01) and increase live birth rate (RD: 0.19, 95% CI 0.07–0.31, *p* = 0.001) in patients with premature ovarian insufficiency [80].

There is also a possibility of dual-double stem cell therapy, which integrates mesenchymal stem cells and hematopoietic stem cells. Such therapy may improve folliculogenesis, normalize hormone serum levels and achieve a successful pregnancy in patients with premature ovarian insufficiency. Clinical studies have also shown superior potential effects of dual-double therapy compared to MSCs or HSCs monotherapies [67].

A pilot study on autologous stem cell ovarian transplantation (ASCOT) procedure was recently reported [81].

The ASCOT procedure consists of the mobilization of bone marrow-derived stem cells (BMDSCs) into peripheral blood using granulocyte-colony stimulating factor (G-CSF) treatment, collection of the cells by apheresis and infusion into the ovarian artery by intra-arterial catheterization on one side. In circulation such cells secrete growth factors such as fibroblast growth factor (FGF)-2, platelet-derived growth factor (PDGF)-BB, thrombospondin (THSP)-1, and insulin-like growth factor (IGF)-1. The authors of the mentioned pilot study reported that the ASCOT procedure improved anti-Mullerian hormone serum levels, increased the total number of antral follicles, the number of antral follicles in the infused ovary, and the total number of follicles measuring >16 mm (*p* = 0.036, *p* = 0.026, and *p* = 0.05, respectively), as well as the number of retrieved oocytes after ovarian stimulation and fertilization rate. The authors concluded that the ASCOT procedure optimized the mobilization of the existing ovarian reserve. Follicular growth waves and spontaneous pregnancies were observed up to six months after the ASCOT procedure, confirming that such a procedure might restore folliculogenesis [81,82]. The authors of the study reported that five pregnancies were achieved during the 6-month follow-up period (three of them by natural conception): one patient delivered twice, another delivered once and a third patient had two miscarriages. The authors of the study concluded that the ASCOT procedure could be an alternative before oocyte donation for women with POI [81]. However, adequately powered clinical studies are still needed.

There are complications associated with autologous stem cell therapy such as immune reactions due to improper handling during isolation and re-implantation or potential tumor formation associated with cultured MSCs: undifferentiated cells are multipotent and may result in neoplasia [83].

Other pioneering studies have evaluated the use of gamete formation from stem cells and isolation of ovarian stem cells. Ovarian stem cells can be found in the ovarian cortex in pre- and postmenopausal women and are hypothetically capable of replenishing the ovarian reserve in relation to oocyte depletion [84].

Ovarian stem cells are smaller than mature oocytes, usually <10 μm in diameter, and during an in vitro three-week culture they gradually enlarge into round-shaped cells with a large nucleus achieving a diameter of 50–70 microns or more, becoming oocyte-like cells. It has been proved that such cells are haploid and the authors of the study concluded that such oocyte-like cells are apparently suitable for IVF [85]. Still, there are no adequate human studies on this issue. On the other hand, there are unsolved questions regarding whether manipulation induces biologic alterations in maturing oocytes derived from ovarian stem cells, and further work is needed before ovarian stem cell technology can be used in clinical application.

### 5.3. In Vitro Activation of Dormant Follicles

It was estimated that after menopause, there are still 1000 follicles in the ovaries [86].

The ovaries of patients with premature ovarian insufficiency also contain dormant primordial follicles capable of being activated in vitro. Recently, primordial follicle activation was successfully used in human fertility treatments and live births occurred [87].

The first clinical protocol of in vitro activation (IVA) of dormant follicles was developed by Kawamura et al. [88]. A laparoscopic ovariectomy was performed in 27 patients with POI, where the ovarian cortex was cut into strips (1–2 mm thickness and 1 × 1 cm) and vitrified. Histological analyses of randomly selected strips revealed that the ovaries of 13/27 patients contained residual follicles. The frozen ovarian strips were thawed and fragmented into cubes of 1–2 mm^2^, treated with Akt-stimulating drugs for two days and auto-transplanted by laparoscopy beneath the serosa of the fallopian tube. The serosa of the fallopian tube was chosen due to its good vascularization. Ultrasound monitoring and estradiol measurements once or twice a week detected follicular growth in eight patients. When the antral follicles reached 5 mm in diameter, daily FSH stimulation was performed, followed by HCG administration when a follicle reached >16 mm in diameter. Oocyte retrieval was performed by transvaginal ultrasound-guided aspiration. Mature oocytes were found in five patients and intracytoplasmic sperm injections (ICSI) were performed using the husband’s sperm. There were six embryos from three patients, which were cryopreserved at the four-cell stage. The result included a biochemical pregnancy in one patient, and in another patient a clinical pregnancy resulting in the live birth of a healthy male baby at 38 weeks of pregnancy.

Ten years ago, Suzuki et al. reported the results of the study involving ten new patients with premature ovarian failure treated with the same procedure [89]. The study included 37 patients, where 54% had residual follicles based on their histology. Follicle growth in autografts occurred in 9/20 patients, and 24 oocytes were retrieved from six of the patients. IVF/ET was performed in four patients; three pregnancies were achieved (one miscarriage and two live births). Still, further studies are needed to identify the best place for ovarian auto-grafting or biological markers to indicate the presence of residual follicles in patients with premature ovarian insufficiency. The authors of the study also proposed that one ovary should be cryopreserved at an earlier stage of POI, which provides a better chance of preserving more ovarian follicles and provides another non-invasive treatment option for infertility. This is logical because premature ovarian insufficiency is a gradual process and the number of follicles decreases with years.

The authors of another study also reported the successful treatment of premature ovarian insufficiency using the IVA technique in 14 patients who received routine hormonal treatments with no follicle development before the IVA treatment. The authors applied a similar IVA procedure as described, using fresh ovarian tissue instead of frozen thawed tissue. They also simplified grafting by using an applicator (endometrial sampling device) to transplant multiple ovarian tissue cubes beneath the serosa of both fallopian tubes. During the one-year follow-up period authors reported that six of the 14 patients showed 15 follicular development waves reaching the preovulatory stage, and six oocytes were obtained from five follicular waves. Follicular waves were either spontaneous (occurring from 20 days to 10 months after grafting) or stimulated with HMG alone or in combination with a GnRH agonist. There were 11 spontaneous follicular waves in four patients, resulting in three successful oocyte retrievals. There were four HMG-induced follicular waves with two successful oocyte retrievals, all in patients treated with HMG and the agonist. One healthy baby was delivered after fresh embryo transfer and there were three frozen embryos remaining [90].

After the first orthotopic auto-transplantation of ovarian tissue more than 40 babies were born. Heterotopic transplantation was also performed, but there is a possibility that it may not provide an optimal environment for follicular development. Allotransplantation has been successfully attempted in monozygotic twins and genetically different sisters [86,91].

The exact mechanism of dormant follicle activation is not known. Animal studies demonstrated that the loss of function of any of the inhibitory molecules for follicular activation leads to premature and irreversible activation of the primordial follicle pool and the consequence is the exhaustion of the resting follicle reserve, resulting in premature ovarian insufficiency in mice [92].

Excessive activation of the primordial follicle pool could also lead to premature ovarian insufficiency in humans [22] and a number of intraovarian factors including growth factors such as vascular endothelial growth factor (VEGF), basic fibroblast growth factor, and keratinocyte growth factor are responsible for primordial follicle activation from a dormant state.

On the other hand, AMH is known as an inhibitor of primordial follicle recruitment and it decreases the sensitivity of follicles to FSH-dependent selection for dominance [93].

The occurrence of spontaneous follicular growth without exogenous gonadotropin stimulation indicates that IVA might have potential in the treatment of all aspects of ovarian premature insufficiency, but adequate clinical studies regarding long term risks of the procedure should be performed [94].

### 5.4. Platelet-Rich Plasma Treatment

Platelet-rich plasma (PRP) is a fraction of autologous blood plasma enriched with platelets at a concentration that is 3 to 5 times greater than the physiological concentration of thrombocytes in whole blood [95].

Platelet-rich plasma (PRP) treatment in premature ovarian insufficiency is based on the presence of different growth factors and their effects on ovarian tissue. Platelets contain growth factors such as platelet-derived growth factor—PDGF, insulin-like growth factor—IGF, vascular endothelial growth factor—VEGF, fibroblast growth factor—FGF, and transforming growth factor—TGF-β, as well as coagulation factors and differentiation factors [96]. These factors are involved in angiogenetic, immunosuppressive and regeneration processes. The use of PRP induces angiogenesis and increases blood supply, stimulating follicular recruitment, proliferation and differentiation of cells [97].

VEGF is involved in neovascularization, and alteration in VEGF content might be a significant factor in compromised folliculogenesis [98].

Another growth factor, PDGF, is also included in angiogenesis, promoting endothelial cell proliferation [99].

TGF-β inhibits macrophage and lymphocyte proliferation and regulates the mitogenic effects of other growth factors [86,100].

IGF can reduce the expression of apoptosis [101].

The explanation of the PRP treatment mechanism on the ovaries is based on the theory that renewable ovarian stem cells exist in adults and that such cells may be utilized as an oocyte source for women seeking to extend their fertility. There is a hypothesis that the growth factors from PRP injected inside the ovaries interact with receptors to influence cell differentiation. PRP treatment could enhance ovarian function by stimulating dormant follicles and inducing the differentiation of potential ovarian stem cells into new young oocytes [102,103]. Nevertheless, this is still a theory.

Ovarian treatment with PRP improved serum levels of FSH, LH, estradiol and AMH levels and enabled spontaneous pregnancies and better IVF outcomes. The authors of a recent systematic review and meta-analysis based on 38 studies involving 2256 women with diminished ovarian reserves reported that PRP treatment resulted in a statistically significant improvement in the main fertility parameters [104]. Serum AMH levels significantly increased from a mean difference (MD) of 0.20 ng/mL (n = 856, *p* > 0.001, 95% CI: [0.12; 0.28]) in the first month after PRP treatment to a MD of 0.36 ng/mL three months after the therapy (n = 881, *p* = 0.002,95% CI: [0.20; 0.52]). Serum FSH levels decreased significantly from one month after PRP treatment: the mean difference MD of −10.20 mIU/mL (n = 796, *p* > 0.039, 95% CI: [−19.80; −0.61]) decreased to −8.87 mIU/mL after 3 months (*p* = 0.010, 95% CI: [−14.19; −3.55]). LH serum levels also decreased every month after PRP treatment, but the change did not reach a statistical level of significance. The authors of the meta-analysis also reported improvements of three main parameters characterizing the dynamics of IVF treatments: the antral follicle count (AFC), the number of retrieved oocytes, and the number of embryos created. The post-treatment antral follicle count was higher by 1.60 follicles compared with the baseline, and the difference was significant (*p* = < 0.001, 95% CI: [0.92; 2.27]). Significantly more oocytes were retrieved after PRP treatment, with a mean difference of 0.81 more oocytes retrieved (*p* = 0.002, 95% CI: [0.36; 1.26]). The incidence of spontaneous pregnancy following PRP treatment showed a rate with a proportion of 0.07 (n = 1370, 95% CI: 0.04–0.12), the rate of biochemical pregnancy was 0.18 (n = 1800, 95% CI: 0.15–0.22), and the live birth rate was 0.11 (n = 1482, 95% CI: 0.07–0.15) (Table 2) [104]. Nevertheless, the authors concluded that further multicenter, randomized trials with large patient populations and a longer follow-up period are needed to certify their results and to develop the most effective treatment protocol.

A combination of autologous in vitro ovarian activation with stem cells and autologous growth factors was also described [105,106]. The authors of the study involving 50 patients with premature ovarian insufficiency applied the SEGOVA method, a group of procedures which offers minimally invasive approaches including ovarian cortex laparoscopic biopsy and re-transplantation through ultrasound control to the same site from which the tissue was taken for in vitro activation. Activation of the obtained ovarian tissue is performed using autologous platelet-rich plasma growth factors instead of the chemical stimulation of genetic pathways. This procedure also involves the application of bone marrow stem cells for transplantation in patients with premature ovarian insufficiency together with cytokines that have antiapoptotic, antifibrotic, anti-inflammatory and immunoregulatory effects. The authors of the study reported beneficial effects on hormonal serum levels and an increased number of ovarian follicles: during the twelve-month follow-up period after re-implantation 32/50 patients (64% of patients) had follicles. IVF was performed, resulting in aspirated oocytes in 8/50 patients (16% of the total number of patients) or 8/32 women with follicles (25% of the follicle positive patients). The fertilization rate was 75%, so 6/32 had embryos, and embryo transfer was performed in 4/32 women with follicles. One patient conceived with IVF, giving birth to twin babies. Two patients spontaneously conceived after transplantation [105]. The main advantages of such a procedure are a conservative minimally invasive surgery (ovarian cortex laparoscopic biopsy), the use of autologous platelet-rich plasma growth factors instead of chemical agents for activation of ovarian tissue, the use of ultrasound-guided re-transplantation of activated ovarian tissue and avoiding the second laparoscopy. There is also an advantage of autologous transplantation in avoiding all transplant rejections that can complicate allogenic transplantation. Autologous stem cell therapy is performed without incubation, thereby avoiding all complications due to long-term incubation of stem cells in substrates and a possibility of losing their function as well as a possibility of unstable DNA.

All of the mentioned treatments designed to improve fertility in patients with POI are not currently supported by any national or international guidelines. There is no reliable clinical evidence for their effectiveness. Such treatments are still experimental therapies and are not currently recommended for clinical use outside of a research setting. Nevertheless, it is worth mentioning these attempts.

A summary of the described newly developed methods is presented in Table 2.

### 5.5. Treatment of Underlying Autoimmune Disorders in Infertile Patients with POI

The treatment of underlying diseases in patients with POI with autoimmune disorders, such as thyroid-related disturbances, Addison’s disease, diabetes mellitus type 1, SLE, etc. (Table 1), are essential before the treatment of infertility and possible pregnancy. Such autoimmune disorders often represent a spectrum of immune disturbances that lead to decreased fertility and an increased risk of pregnancy loss. It was reported that the presence of autoimmune thyroid diseases (ATD) impairs the outcome of COH in ART cycles in women with a good ovarian reserve, while the probability of a poor response to COH is high and independent from ATD in women with low serum AMH levels [107]. The authors of a meta-analysis including 17 studies addressing the effect of thyroid autoimmunity on ovarian reserve/response and on assisted reproductive technology (ART) outcomes concluded that many questions remain unanswered and the decision for treatment should be based on the current recommendations and individualized clinical judgment on the cause of the infertility as well as on the obstetric history of the women [108].

Immunosuppressive therapy with glucocorticoids is an optional treatment in a selected population of well-defined autoimmune POI and in patients with idiopathic POI, in whom the resumption of ovarian activity is possible [109]. It was reported that a short course of glucocorticoid suppression (2 mg dexamethasone for 4 weeks prior to a COH cycle) resulted in the retrieval of a good number of oocytes and a live birth following a thawed embryo transfer in a patient with POI and autoimmune polyglandular syndrome type 2 (primary adrenal insufficiency, autoimmune thyroid disease, and type 1 diabetes mellitus) [110].

## 6. Discussion

### 6.1. The Course of POI

Regarding the hypothesis of molecular mechanisms involved in the development of spontaneous premature ovarian insufficiency, it seems that the success of infertility treatment essentially depends on the timing of the treatment initiation.

Several factors have been used to predict menopause and POI such as age, anti-Müllerian hormone (AMH), inhibins, follicle-stimulating hormone serum levels, antral follicle counts (AFC), menstrual cycle length, and, recently, some genetic markers. It seems that age has the best predictive power and all the other factors are only adding to the prediction of menopause in a very limited way [111].

On the other hand, there is no established method to exactly measure the remaining follicle pool; moreover, there is no proven method which can determine that a woman has no primordial follicles remaining in the ovary. Serum AMH level is considered to be the most reliable method to determine the ovarian reserve [6]. AMH levels below 8 pmol/L (1.12 ng/mL) have shown high sensitivity (85%) and specificity (100%) for diagnosing POI in women with secondary oligomenorrhea [36]. Low levels of AMH in young women indicate a greater risk for POI and could facilitate an early diagnosis [111].

Nevertheless, current guidelines do not recommend AMH testing to predict POI due to insufficient evidence of accuracy [6]. From a clinical point of view, only the lowest serum AMH levels reliably predict the development of POI.

It seems logical that a diminished ovarian reserve precedes complete follicle depletion. In fact, POI is a continuum and patients may move from one state to another. Some authors distinguish clinical states of occult, biochemical and overt ovarian insufficiency to describe the clinical development of non-iatrogenic POI prior to definitive follicle depletion [112,113].

Initially, there is occult ovarian insufficiency presenting as unexplained infertility in patients with normal serum FSH levels, but with an inexplicable failure to respond adequately to FSH therapy during controlled ovarian hyperstimulation (COH) for ART. The next clinical stage is biochemical primary ovarian insufficiency presenting as infertility in patients with an elevated basal serum FSH level and the patients also fail to respond adequately to COH. The next stage in the continuum is overt ovarian insufficiency in infertile patients with elevated basal serum FSH levels in association with disordered menstrual cycles (oligomenorrhea, polymenorrhea, or metrorrhagia). The last stage in the continuum is an extreme state of complete primordial follicle depletion characterized by the presence of amenorrhoea, menopausal gonadotropin serum levels and permanent infertility.

There is a delay in the diagnosis and treatment of POI due to existence of an occult stage and many patients lose the opportunity for more successful infertility treatment. One study reported a mean delay of 4.4 years in the diagnosis of POI after the onset of menstrual irregularity and such patients had not taken any hormone replacement therapy for 2.8 years. The authors of the study explained this long period without any treatment with a variety of reasons such as nonadherence, delay in diagnosis and confusion regarding the use of hormonal therapy [114].

### 6.2. Spontaneous Reversion of POI

Regarding future fertility, iatrogenic ovarian insufficiency is a definitive state in most of the cases (i.e., after surgery) and for these patients oocyte donation is currently the only realistic option.

On the other hand, there is a possibility of spontaneous restoration of ovarian function in patients with non-iatrogenic or spontaneous ovarian insufficiency. In some cases, this may occur years after the diagnosis has been established. Many women with a clinical diagnosis of spontaneous premature ovarian insufficiency still have dormant follicles and the presence of antral follicles has been reported in more than 70% of patients with POI [35,115,116,117].

Clinical practice has shown that it might be possible to change or reverse the factors responsible for spontaneous premature ovarian insufficiency, allowing the spontaneous restoration of ovarian function and even pregnancy. The authors of a systematic review including 52 case reports, eight observational studies, nine uncontrolled studies and seven controlled trials reported that patients with spontaneous premature ovarian insufficiency have a chance of between 5 and 10% of getting pregnant, with the birth of a healthy child occurring in approximately 80% of such pregnancies [118].

The authors of another study including 358 patients with premature ovarian insufficiency reported that twenty-one spontaneous pregnancies (16 births, five miscarriages) occurred in 15 (4.4%) patients [119]. This is the consequence of a residual ovarian follicle pool and restored ovarian function which is a unique characteristic of premature ovarian insufficiency. This is also the main distinction from an early menopause, defined as the cessation of menstrual cycles between the ages of 40 and 45 years [33].

Such a restoration may happen years after the diagnosis of premature ovarian insufficiency was established [35]. The authors of the study exploring the clinical and endocrine characteristics of idiopathic premature ovarian failure in 26 patients reported that nine of the 18 patients showed hormonal evidence of functioning ovarian follicles, and four out of nine women had viable oocytes on biopsy; ovulation was noted in five patients and spontaneous pregnancy occurred in one [35].

The authors of another study, including 65 patients with karyotypically normal spontaneous premature ovarian failure, reported that some women have ovarian follicles that function intermittently: during follow-up 50% of women demonstrated ovarian function (estradiol serum levels > 183 pmol/L or 50 pg/mL), but only 16% achieved an ovulatory serum progesterone level. Such Graafian follicles capable of responding to FSH, which has elevated levels in patients with POI, are also under the influence of high LH serum levels. The consequence is premature luteinization. Ultrasound examinations revealed antral follicle in 40% of patients. In six patients follicle biopsy was performed and luteinized Graafian follicles were found. The authors concluded that some antral structures seen on the ultrasound examination were luteinized follicles and inappropriate luteinization is the major pathogenetic mechanism of spontaneous premature ovarian insufficiency in patients with a normal karyotype [120].

According to the mentioned reports, it is likely that spontaneous premature ovarian insufficiency is not a definitive state, at least in some patients. Premature ovarian insufficiency “does not mean permanent cessation of ovarian function” [33]—a statement which gives hope to patients regarding spontaneous remission and subsequent pregnancy. Unfortunately, there are no proven treatments that could help the development of such a restoration of ovarian function. It was reported that ethynyl estradiol in a dose of 0.02 mg was successful in the induction of ovulation in such patients, and even pregnancy was achieved [121].

Another case report demonstrated the ability of ethynyl estradiol to lower elevated serum FSH levels in patients with imminent ovarian failure and tubal factor infertility. This effect of ethynyl estradiol allowed restoration of down-regulated FSH receptors and improved responsiveness to endogenous gonadotropins. IVF/ET was performed, one oocyte was retrieved and fertilized, one embryo was transferred and the patient conceived [122].

The authors of the case report regarding the beneficial effects of lowering elevated serum FSH levels with ethynyl estradiol and the outcome of gonadotrophin stimulation for IVF, which resulted with pregnancy, concluded that high serum FSH levels in a woman of younger reproductive age does not mean egg quality is poor [123].

The authors of the study regarding the effects on fertility which included 44 patients with premature ovarian insufficiency treated with combined estrogen–progesterone treatment combined with daily supplementation of 25 mg of micronized oral dehydroepiandrosterone (DHEA) and melatonin supplementation in a daily dose of 3 mg concluded that such a combination could optimize fertility and lead to successful pregnancy in patients with POI: there have been 13 completed term pregnancies so far with normal fetal growth and development [124].

Our own clinical practice demonstrated the beneficial effects of estradiol, followed by progesterone for luteal support, on the restoration of ovarian function in a patient with premature ovarian insufficiency who spontaneously conceived after one year of such therapy. Unfortunately, the pregnancy was ectopic. Still, there is also a possibility for the spontaneous reversion of some etiological factors or mechanisms responsible for premature ovarian insufficiency. Nevertheless, adequate studies on this issue are still lacking.

Naturally, no one could lean on the possibility of spontaneous pregnancies in such patients and wait, so other described methods are currently developed for the treatment of infertility in patients with spontaneous ovarian insufficiency. The problem with such methods is a manipulation of the ovarian tissue and oocytes and exposure to chemical agents in culture, unstable DNA during long-term culture and possible long-term effects. It is also hard to obtain an adequate number of ovarian follicles for in vitro activation. Moreover, it is not possible to adequately compare the results of different studies due to variable protocols used for stem cell treatments and PRP. The majority of such studies have included a small number of patients, and even the diagnostic criteria applied to establish the diagnosis of premature ovarian insufficiency (i.e., the serum FSH levels, age of the patients, etc.) vary between the studies. After all, regarding the possibility of spontaneous pregnancies, it is difficult to distinguish between the possible success of the treatment and the spontaneous restoration of POI. There is a need for adequate, multicentric studies to solve the issues mentioned.

According to reported studies platelet-rich plasma therapy is the most frequently used method for infertility treatment in patients with POI, currently being the most realistic option for routine clinical practice. Nevertheless, in vitro activation of dormant follicles and tissue auto-transplantation represent a logical attempt to use the remaining follicles. Moreover, such methods offer the possibility of spontaneous or induced waves of follicular growth to patients with POI, as well as the SEGOVA method and the ACSOT procedure. The use of the mentioned methods in routine clinical practice depends on the ongoing development of biotechnology, which is unpredictable.

Regarding the possibility of spontaneous restoration of ovarian function in patients with non-iatrogenic ovarian insufficiency, future studies should develop the described methods to identify those patients who still have an adequate number of dormant follicles in their ovaries, possibly with the use of some biochemical markers or another non-invasive method—not the ovarian biopsy. Regarding the treatments directed at restoring the function of the remaining follicles, standard and safe protocols should be developed, especially on PRP and stem cell use. It is also important to clarify the possible long-term risks of such procedures, not only for the patients, but also for the babies born following such procedures.

## 7. Conclusions

Currently, oocyte donation is the standard treatment for patients with POI desiring pregnancy. Recently developed methods for the regeneration of ovarian tissue such as stem cell therapy, platelet-reach plasma therapy and in vitro activation of ovarian tissue are still under investigation. All of the mentioned treatments designed to improve fertility in patients with POI are not currently supported by any national or international guidelines.

## Figures and Tables

**Table 1:** Etiology of premature ovarian insufficiency.

**Non-Iatrogenic (Spontaneous) POI**
- idiopathic POI
- genetic—chromosomal abnormalities:
- chromosomal abnormalities:
- X chromosome: Turner sy, mosaic X-chromosomes aneuploidy, X trisomy, etc.
- autosomal abnormalities: X autosomal translocations in autosomal syndromes:
- pseudo-hypoparathyroidism type 1a,
- blepharophimosis, ptosis, epicanthus inversus (BPES) type 1,
- Perrault syndrome (sensorineural hearing loss)
- genetic abnormalities:
- mutation of FMR1 (fragile X messenger ribonucleoprotein 1),
- mutations of FOXL2 gen (cause BPES type 1),
- mutations of CLPP gene (cause Perrault syndrome), etc.
- autoimmune disorders: - autoimmune thyroid diseases, adrenal insufficiency, type 1 diabetes,
rheumatoid arthritis, psoriasis, systemic lupus erythematosus (SLE), etc.
- autoimmune polyglandular syndrome type 1 (APS-1) or
autoimmune polyendocrinopathy-candidiasis-ectodermal dystrophy (APECED)
- autoimmune polyglandular syndrome type 2 (APS-2)
(Addison’s disease, autoimmune thyroid disease, and type 1 diabetes mellitus)
- infections 2013 mumps
- FSH receptor inactivating mutations
- metabolic or enzyme deficiencies:
- deficiency of 17 hydroxylase
- galactosae-1-phosphate uridyl transferase (GALT)
**Iatrogenic POI** - chemotherapy, radiotherapy, uterine artery embolization or surgery

**Table 2:** Summary of newly developed methods for the treatment of infertility in patients with POI.

Method *	Patients (n)	Follow Up	Pregnancies (n)	Live Births
Stem cell therapy:				
- Herraiz 2018 [81]—ASCOT procedure (BMDSC)	17	6 months	5	3
- Yan 2020 [78]—UCMSC	61		4	4
In vitro activation of dormant follicles:				
- Kawamura et al. 2013 [88]	27		2	1
- Suzuki 2015 [89]	37		3	2
- Zhai 2016 [90]	14	12 months	1	1
Platelet-rich plasma:				
- Éliás 2024 [104] (meta-analysis including 38 human studies):	2256			
- biochemical pregnancy—(analysis included 19 studies)	1800		323	
- spontaneous pregnancy—(analysis included 16 studies)	1370		97	
- live birth—(analysis included 12 studies)	1482			177
SEGOVA method:				
- Tinjić & Ljubić 2021 [105]	50	12 months	3 (1 after IVF, 2 spontaneous)	1

Abbreviations: ASCOT—autologous stem cell ovarian transplantation, BMDSC—bone marrow derived stem cell, UCMSC—umbilical cord-derived mesenchymal stem cells. * case reports are not included in the table.

## Data Availability

The data analyzed during this study are included in the published article and the manuscript conforms with the MDPI editorial policies and ethical policies.
